# The Art of Medicine: “Meeting Patients Where They’re At”

**DOI:** 10.31486/toj.24.5041

**Published:** 2024

**Authors:** Elyse Stevens

**Affiliations:** Department of Internal Medicine, Section of Community and Population Medicine, Louisiana State University Health Sciences Center, School of Medicine, New Orleans, LA

*Mr P is a 56-year-old male with a history of insulin-dependent type 2 diabetes and hypertension complicated by chronic housing instability, food insecurity, and complex undifferentiated mood disorder. He has been staying in a tent in an encampment for the past 8 months since his previous housing arrangement fell through. For several years, he has used local emergency departments for refills of his medications and has presented multiple times for hypertensive and diabetic emergencies. He has begun to develop painful peripheral neuropathy and has noticed his vision is getting worse. He is increasingly concerned about an ulcerating wound on his leg that has become deeper and increasingly painful in the past few months.*

*He has had several negative experiences as a “frequent flyer” in the emergency department. Sleeping on concrete, he has developed chronic back pain that has resulted in a chart littered with “med-seeking” and “malingering.” Unable to get treatment, he has turned to substance use.*

*Mr P has become frustrated with the process. He has missed several appointments to establish primary care. He frequently loses track of the date and day of the week, and he does not have a phone or a watch. He recently acquired a housing voucher but is anxious about leaving his encampment community. He has been trying to manage his chronic health conditions on his own, borrowing medications from friends just to avoid another encounter with the health care system.*

Mr P's case is not uncommon. Patients experiencing housing instability are less likely to seek care for any health concern, less likely to take medications as prescribed, and less likely to follow up in clinic. As a result, chronic health conditions are left untreated; preventive health measures and screenings fall behind; and patients are exposed to risk factors, pathogens, and physical dangers unique to their living conditions. Physical disabilities, untreated mental health conditions, and substance use disorders result from and contribute to an individual's inability to attain and maintain housing stability. This cycle continues indefinitely—sick people become unhoused, unhoused people become sick, and sick people just stay sick.^1^

The city of New Orleans’ most recent housing initiative, a rendition of an evidence-based approach known as Housing First, seeks to interrupt this cycle by relocating individuals from encampments into supportive housing.^2,3^ While we await the results of this initiative, we in the medical community must respond to a call to action from within—firstly, to reflect critically on our contribution to the perpetuation or cessation of this cycle, and secondly, to courageously forge new paths in our practices that intentionally and actively respond to the health disparities in our communities without regard to what we have all been told is “just the way things are.” In New Orleans, a dedicated group of health care volunteers rises to the occasion each week and answer that call to action in full force.

Every Saturday, volunteers from Louisiana State University (LSU) School of Medicine, in partnership with the community health outreach organization Freestanding Communities^4^ (led by local paramedic and educator Aquil Bey), host a free, all-day walk-in clinic on the second floor of the Ozanam Inn. A longstanding pillar of the community, Ozanam Inn is a shelter and resource center for people experiencing homelessness and housing instability.^5^ Ozanam Inn is one of only a handful of such facilities in New Orleans and is unique in its approach, combining structured programming with open doors and inclusivity and valuing empowerment and sustainability on par with the alleviation of immediate human suffering. As a testament to these values, the Ozanam Inn opens its doors every weekend not only to our swarm of volunteers but also to any patient seeking our services.

Since the Saturday Ozanam Clinic reopened post-COVID, patient volume has steadily increased from just 4 or 5 to consistently more than 20, and often close to 30, patients per day. Yet, not insignificantly, we have not produced or distributed a single flier, brochure, or business card for the Saturday Ozanam Clinic in more than a year. Our reputation precedes us and speaks for itself. Patients are referred by neighbors, friends and family, cellmates, case workers, and even by local health systems. Patients come from all over the city—from downtown to uptown to the Lower Ninth Ward and New Orleans East—and even from other cities as far away as Thibodeaux and Baton Rouge.

From fundraising to care delivery, the Saturday Ozanam Clinic, like all sites of the LSU Student Run Community Clinics,^6^ is entirely student run. Elected medical students serve in leadership, dedicating a year of their already tortuous medical education to leading their classmates in hundreds of hours of community service. Ten to 20 medical students and community health advocates arrive at each shift, eager and ready to learn and make a difference.

The Saturday Ozanam Clinic runs with a smoothness that far surpasses that of many primary care clinics, including my own. The community health advocates from Freestanding Communities welcome patients with warmth and kindness as they coordinate their intake and triage. A community health advocate then hands the patient off to the clinic leader, who logs the patient on the tracking board and assigns a care team of 2 to 3 medical students. Care teams conduct a focused history and physical, formulate a plan of care, and present to 1 of 2 to 3 supervising physicians.

Through patient interactions, students learn how to elicit histories on the patients’ terms, how to ask difficult questions, and how to hear difficult responses. Together, we are learning to confront the tragedies of this human condition—how to treat back pain in a patient who sleeps on concrete, edema in a patient who has no bathroom access, and anxiety in a patient who lives in constant peril. We learn to adapt guidelines that were never written for our patient population and to interpret evidence from studies in which our patients were never consulted.

Teaching takes place mainly at bedside, involving patients in every aspect of clinical education and decision-making and modeling for students what a truly patient-centered practice looks like. Much of what we do for patients has little to do with medicine. In fact, the medicine is often the easiest part. Sometimes the most impactful assistance we can provide is by performing tasks such as helping a patient clear 14 voicemails to make room in their inbox for appointment reminders, clipping toenails and cleaning out earwax, fixing broken bicycles and wheelchairs, learning about chair yoga, and even reconnecting patients with long-lost relatives through investigative social media searches.

After each visit, patients are discharged with thorough follow-up plans that are focused on arriving at the next step in treatment, however seemingly small that step is, and starting at wherever the patients may be on their journey. In addition to health outcomes such as quitting smoking, reducing blood pressure, and controlling cholesterol, our clinic focuses on the proximal outcomes: the patient feeling known, respected, involved, engaged, and knowledgeable. These are the outcomes that the care team must earn with sincerity, curiosity, and compassion. The goal is to earn enough trust for a return visit. Even if a single step takes a dozen visits over weeks or months, every step is a success, and eventually we will get where we need to be.

The Saturday Ozanam Clinic is a beacon for patients like Mr P who often find it difficult to follow up in traditional care settings. The reasons for this difficulty are many. While undoubtedly a reflection of the high-quality, low-barrier care that draws patients to the clinic, the success of the Saturday Ozanam Clinic is moreover a commentary on the enormity of the health disparities this community faces, disparities that are widened by our system's adherence to traditional models of care that exclude patients facing socioeconomic hardship, particularly those experiencing homelessness or housing instability.

To begin with, the demand for services simply outweighs availability. Most primary care clinics in the area that accept Medicaid and uninsured patients book out for several weeks. Specialist clinics book out for months. Such scheduling diverts many primary care complaints and urgent but not emergent care patients to emergency departments that are constantly overflowing, driving up wait times and leaving patients feeling disillusioned, overlooked, and still sick.

Accessibility of care is a challenge deeply rooted in the foundations of the American medical system. Requiring appointments and rescheduling for tardiness, while many argue are necessary to maintain order, are unforgiving of transportation delays, communication challenges, and disorganized minds. Systems requiring working phone numbers and addresses where communications can be mailed quite blatantly exclude those without such resources. The clinical environment itself can also serve as a barrier. Uniformed security at entryways, computerized check-in kiosks, and even the emblematic white coat may project professionalism and modernity to some but are intimidating to others.

The most shameful barriers to care are iatrogenic. Widespread stigmatization of various patient stereotypes infects the charts and minds of clinicians at all levels. Frequent hospital encounters earn patients “frequent flyer” flags. Patients with unresolved pain become “med-seeking.” Patients who advocate strongly for themselves are deemed “difficult,” especially if they tend to do so loudly. “Noncompliant” patients leave “AMA” (against medical advice) if their plan does not align with ours. Patients suffering with substance use or mental health disorders are notoriously undertreated, mistreated, and dehumanized in clinical settings at all levels of care.

All of these mechanisms represent a form of institutional violence through which a simple but lethal message is conveyed to the patients who need us most: “You don’t belong here.”

On Saturdays at Ozanam Inn, every patient belongs. Every patient is greeted with warmth and welcoming smiles. Every chief complaint is taken seriously, and every patient is right on time, no matter when or how they arrive. As our patient volume continues to rise, we continue to adapt. Using a collection of flexible contingencies, we continuously reevaluate patient flow throughout the day and respond with changes in room allocation, triaging tiers, and personnel distribution. To date, we have not refused service to any patient, nor do we intend to.

Our students, indeed all of us, are learning to put words into action and to hold ourselves accountable. “Meeting patients where they’re at” is more than a tagline for us; it is an art form. “Harm reduction” is not a strategy, it is a tenet, and “street medicine” describes a location of service, not a novel new practice. We have no “noncompliant” patients, only noncompliant systems; no “difficult” patients, only patients for whom we are having difficulty finding the right plan of care; and all patients are “med-seeking” because they are sick and seeking medicine.

The team at the Ozanam Clinic arrives every Saturday believing to our core that serving our community is our duty and our privilege. We know that this value is shared by many others in our field. In the face of New Orleans’ catastrophic housing crisis and impending mass intervention, we as a medical community have a responsibility to acknowledge with humility the ways in which our systems have contributed to the perpetuation of this cycle of sickness and indigence. We must be ready and willing to answer this call to action and to open our doors and our minds to new possibilities of what patient care can look like.
